# Corrigendum to “Evaluation of the Genotoxic Potential against H_2_O_2_-Radical-Mediated DNA Damage and Acute Oral Toxicity of Standardized Extract of *Polyalthia longifolia* Leaf”

**DOI:** 10.1155/2021/9836793

**Published:** 2021-05-21

**Authors:** Subramanion L. Jothy, Yeng Chen, Jagat R. Kanwar, Sreenivasan Sasidharan

**Affiliations:** ^1^Institute for Research in Molecular Medicine (INFORMM), Universiti Sains Malaysia (USM), George Town, Penang 11800, Malaysia; ^2^Dental Research & Training Unit, and Oral Cancer Research and Coordinating Centre (OCRCC), Faculty of Dentistry, University of Malaya, Kuala Lumpur 50603, Malaysia; ^3^Nanomedicine-Laboratory of Immunology and Molecular Biomedical Research (LIMBR), School of Medicine (SoM), Faculty of Health, Institute for Frontier Materials (IFM), Deakin University, Waurn Ponds, Geelong, VIC 3217, Australia

In the article titled “Evaluation of the Genotoxic Potential against H_2_O_2_-Radical-Mediated DNA Damage and Acute Oral Toxicity of Standardized Extract of *Polyalthia longifolia* Leaf ” [1], the incorrect representative image for Figure 1(e1) sample was included as noted on PubPeer [2]. The published Figure 1(e1) depicted the histological details of the untreated control rat heart organ. No pathologies were recorded in the histological sections of the heart of the untreated control rat group. Due to an error that occurred inadvertently at the time of figure assembly by the first author, the incorrect representative image for Figure 1(e1) was included. In the current figure, Figure 1(e1) belongs to Figure 1(e2) panel heart images of *P. longifolia* leaf extract-treated rats group. With the agreement of the handling editor, the corrected version of Figure 1(e1) is provided below. The authors confirm that this error did not change the scientific conclusions of the published article and apologize for this human error.

## Figures and Tables

**Figure 1:**
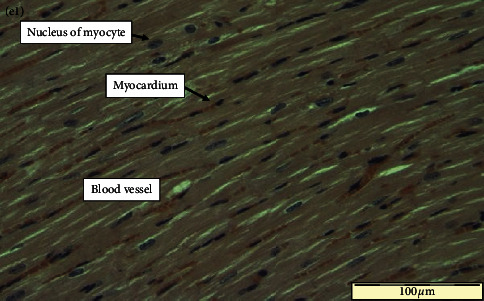
Representative histological photomicrographs of (e1) heart of control group.
